# Dual ligand/receptor interactions activate urothelial defenses against uropathogenic *E. coli*

**DOI:** 10.1038/srep16234

**Published:** 2015-11-09

**Authors:** Yan Liu, Sylvie Mémet, Ricardo Saban, Xiangpeng Kong, Pavel Aprikian, Evgeni Sokurenko, Tung-Tien Sun, Xue-Ru Wu

**Affiliations:** 1 Department of Urology, New York University School of Medicine; 2 Centre d’Immunologie de Marseille-Luminy, Aix-Marseille University, UM2, Institut National de la Santé et de la Recherche Médicale (Inserm), U1104 and Centre National de la Recherche Scientifique (CNRS) UMR7280, Marseille, France; 3 Research and Postgraduation of University Anhembi Morumbi, S. Paulo, SP 03164-000, Brazil; 4 Departments of Biochemistry and Molecular Pharmacology, New York University School of Medicine; 5 University of Washington, Seattle, WA 98195; 6 Departments of Cell Biology, New York University School of Medicine; 7 Departments of Dermatology, New York University School of Medicine; 8 Department of Pathology, New York University School of Medicine; 9 Departments of Veterans Affairs New York Harbor Healthcare System, Manhattan Campus, New York, NY 10010.

## Abstract

During urinary tract infection (UTI), the second most common bacterial infection, dynamic interactions take place between uropathogenic *E. coli* (UPEC) and host urothelial cells. While significant strides have been made in the identification of the virulence factors of UPEC, our understanding of how the urothelial cells mobilize innate defenses against the invading UPEC remains rudimentary. Here we show that mouse urothelium responds to the adhesion of type 1-fimbriated UPEC by rapidly activating the canonical NF-κB selectively in terminally differentiated, superficial (umbrella) cells. This activation depends on a dual ligand/receptor system, one between FimH adhesin and uroplakin Ia and another between lipopolysaccharide and Toll-like receptor 4. When activated, all the nuclei (up to 11) of a multinucleated umbrella cell are affected, leading to significant amplification of proinflammatory signals. Intermediate and basal cells of the urothelium undergo NF-κB activation only if the umbrella cells are detached or if the UPEC persistently express type 1-fimbriae. Inhibition of NF-κB prevents the urothelium from clearing the intracellular bacterial communities, leading to prolonged bladder colonization by UPEC. Based on these data, we propose a model of dual ligand/receptor system in innate urothelial defenses against UPEC.

Epithelial cells covering the mucosal surfaces are in constant interactions with a broad range of microbes, most of which not only pose no threat but provide beneficial effects to the host^1^,^2^,^3^. These so-called “commensal” microbes can form symbiotic relationships with the mucosal epithelial cells by supplying nutrients and keeping out harmful microbes. Other microbes, however, can be pathogenic causing disruption of the anatomic integrity and/or the physiological functions of the epithelial cells and leading to mucosal inflammation and infection. Mounting evidence suggests that the innate immune status of the host epithelial cells plays a key role in distinguishing the commensal microbes from the pathogenic ones^3^,^4^. At the center of this effect is the nuclear factor-κB (NF-κB) family of transcriptional factors that are constitutively expressed by, but normally kept inactive in, the mucosal epithelial cells^5^,^6^. It has recently been shown in the gastrointestinal tract that a basal-level activation of NF-κB, presumably elicited by the commensal gut microbes, is essential for the development, self-renewal, and the absorptive and barrier functions of the intestinal epithelium^7^. On the other hand, when faced with pathogens, the intestinal epithelium can mount robust innate immune responses by markedly activating the NF-κB pathway, leading to the secretion of pro-inflammatory cytokines and recruitment of inflammatory cells. Indeed, reduced responses in this pathway in genetically engineered mice lacking key NF-κB pathway components can result in persistent inflammatory or infectious states^8^. A balanced response by the NF-κB pathway is therefore vital for the intestinal epithelial homeostasis and defenses against pathogens, although whether these responses are as fine-tuned in other mucosal epithelia is considerably less clear.

The mammalian urothelium covers the mucosal surfaces of much of the urinary tract including proximal urethra, bladder, ureters and renal pelvis and, as such, is at the forefront of interacting with microbes that have gained access into the urinary tract^9^,^10^. Although comprised of a single cell type, i.e., urothelial cells, urothelium can be morphologically subdivided into distinct cell layers based on their degree of differentiation. The basal layer, in contact with the basement membrane, is the least differentiated, containing keratin 5/14- and p63-positive cells that are the likely source for urothelial renewal^11^,^12^. The intermediate layer is moderately differentiated and varies in thickness depending on the species (1 layer in mice and 3–5 layers in humans). The superficial layer consists of highly flattened (squamous) and terminally differentiated umbrella cells that produce a copious amount of integral membrane proteins called uroplakins (Ia, Ib, II, IIIa and IIIb)^13^,^14^,^15^,^16^,^17^,^18^. Along with the tight junction adjoining the umbrella cells and the lipid bilayer, the uroplakins form the apical surface of the urothelium that constitutes the most effective permeability barrier in the body^19^,^20^,^21^. Of all mature uroplakins, only uroplakin Ia carries unmodified terminal mannoses that specifically bind the FimH adhesin of type 1-fimbriated uropathogenic *E. coli* (T1F-UPEC)^22^,^23^,^24^,^25^, the etiological agent of up to 85% of all uncomplicated urinary tract infections^26^,^27^,^28^. Once bound to the urothelial surface, T1F-UPEC re-organizes the plasma membrane and cytoskeletons of the umbrella cells, gaining entry into their cytoplasm to multiply and form so-called intracellular bacterial communities (IBCs^29^,^30^). The IBCs not only are protected from antibiotics and host immune attacks, but can break out of the umbrella cells to seed a fresh round of infection or invade into deeper urothelial layers to become quiescent reservoirs for chronic, recurrent infections^29^,^30^.

Despite the notable progress in chronicling the lifecycle of T1F-UPEC inside the urothelium, the positive role or the dispensability of NF-κB in countering the invading bacteria and how this might affect the pathogenesis of urinary tract infection remain unclear. This is in part due to the fact that previous reports primarily employed cell lines derived from advanced human bladder cancers (e.g., T24, 5637, etc.)^31^,^32^,^33^,^34^. On the one hand, incubation of these cell lines with lipopolysaccharide (LPS) or UPEC strains triggered nuclear translocation of NF-κB and increased production of inflammatory cytokines, suggesting that NF-κB is activated by UPEC^32^,^35^,^36^. It could be argued, however, that like many other cancer types the NF-κB pathway is constitutively over-activated in bladder cancer cells^37^,^38^, thus representing a heightened activation state that may not reflect how NF-κB acts in normal urothelial cells. Additionally, bladder cancer cell lines are often mono-layered and lack appreciable amounts of uroplakin proteins or bona fide umbrella cells, as they comprise primarily undifferentiated cells equivalent to basal and/or intermediate cells of the *in vivo* urothelium^39^,^40^,^41^,^42^. Thus, responses of cultured bladder cancer cells to UPEC could deviate significantly from those of the normal urothelium *in vivo*. On the other hand, other reports described that T1F-UPEC strains can profoundly suppress the LPS-induced NF-κB activation^31^,^43^. Since inhibition of NF-κB can promote apoptosis^44^, these results seemed in accordance with earlier observations in mouse models showing that T1F-UPEC infection increased urothelial apoptosis^45^. Taken together, the existing data are somewhat conflicting and the fundamental question of whether T1F-UPEC activates or inhibits the NF-κB pathway in urothelial cells *in vivo* remains unresolved.

We undertook this study to determine whether *in vivo* challenge of normal urothelium by T1F-UPEC activates or inhibits the NF-κB pathway using a novel panel of *in vivo*, physiologically relevant systems including NF-κB transgenic reporter mice, wild-type mice and NF-κB pathway-deficient mice. We examined (i) whether the adhesion of UPEC to their urothelial receptors *in vivo* is a prerequisite for altering the activation status of NF-κB, (ii) whether distinct urothelial layers respond differently to T1F-UPEC, (iii) whether the multiple nuclei within a single umbrella cell react synchronously or non-synchronously to T1F-UPEC infection, (iv) whether NF-κB activation is dependent on Toll-like receptor 4 signaling and (v) whether inhibiting NF-κB affects the outcome of urinary tract infections. Our results provide the first *in vivo* evidence establishing that NF-κB is strongly activated by T1F-UPEC in multinucleated urothelial umbrella cells via a dual ligand/receptor system, i.e., the FimH/UPIa and the lipopolysaccharide/TLR4. Our data underscore the critical importance of the activation status of urothelial NF-κB in affecting the disease course and clinical manifestation of microbe-related urinary tract conditions including acute cystitis, recurrent bladder infections, pyelonephritis, asymptomatic bacteriuria and normal microbiome of the healthy urinary tract.

## Results

### Urothelial Adhesion of Type 1-fimbriated Uropathogenic *E. coli* Markedly Activates the Canonical NF-κB in Superficial Umbrella Cells

**Figure 1:**
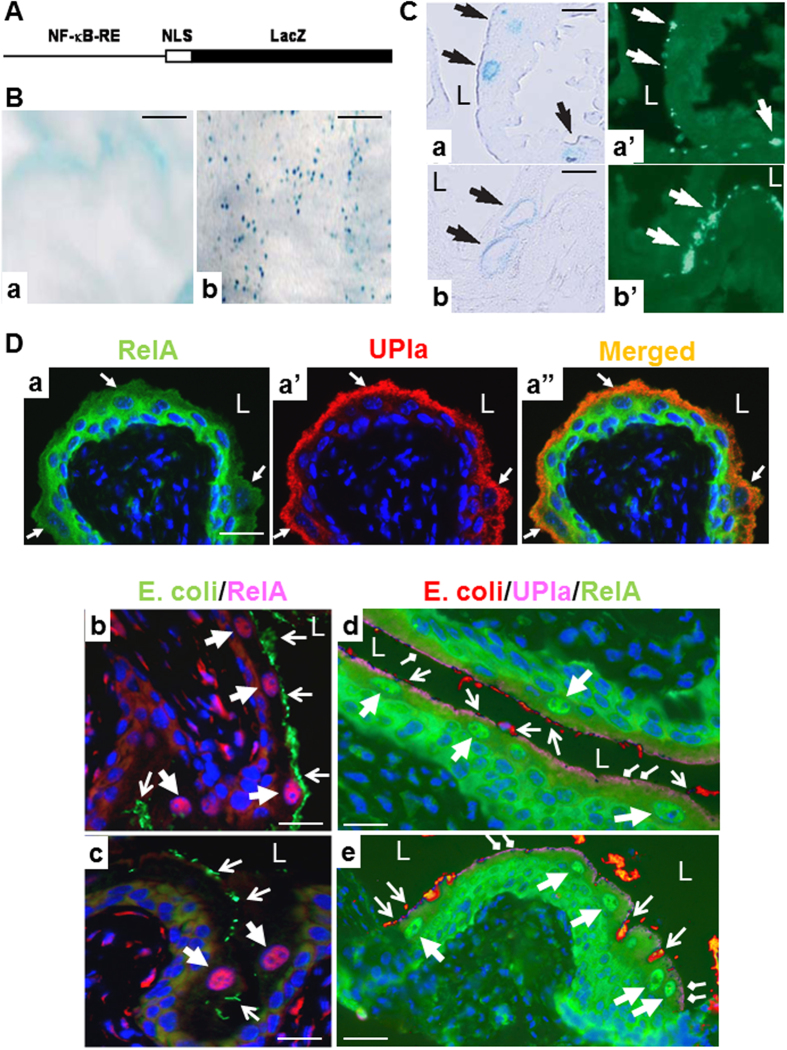
T1F-UPEC triggered urothelial NF-κB activation in transgenic reporter mice and wild-type mice. (**A**) The reporter mice harbored a transgene consisting of NF-κB responsive elements (RE) that controlled the expression of *lacZ* reporter gene fused in-frame at the 5′-end to a nuclear localization sequence (NLS)^46^,^47^,^48^. (**B**) NF-κB-RE/NLS-*lacZ* transgenic mice were transurethrally administered with PBS (**a**) or that containing T1F-UPEC strain UTI89 (10^8^ cfu) (**b**). The urinary bladders were excised and stained *in situ* for β-galactosidase activity and photographed with a dissecting microscope. Note only slight, diffuse background in a PBS-instilled bladder (**a**) and the strong, blue-dotted staining in T1F-UPEC-instilled mice (**b**). (**C**) T1F-UPEC instilled, NF-κB-RE/NLS-*lacZ* transgenic mouse bladders were subject to frozen sectioning and histochemical staining for β-galactosidase activity. The adjacent sections were stained with an anti-*E. coli* antibody followed by an FITC-conjugated secondary antibody. Note the positive β-galactosidase activity of urothelial umbrella cells (a and b; black arrows) and the surface-attached *E. coli* (a’ and b’; white arrows). L denotes bladder lumen. (**D**) Subcellular localization of RelA subunit of NF-κB at the steady state and upon T1F-UPEC infection in wild-type mouse urothelia. Mice were transurethrally instilled with PBS (**a**) or T1F-UPEC strain UTI89 (10^8^ cfu) (**b–e**). One-hour after instillation, the urinary bladders were processed for paraffin embedding/sectioning and then immunofluorescent staining using primary antibodies as indicated, followed by fluorescein-conjugated secondary antibodies. Note the exclusive cytoplasmic staining of RelA in all urothelial layers in a PBS-instilled bladder (a and a”). Arrows mark the apical surface (positive for uroplakin Ia (UPIa)) of the urothelium. L denotes the bladder lumen. Also note, in UTI89-instilled bladders, prominent nuclear translocation of RelA mainly in the urothelial umbrella cells (b and c; thick arrows) over which many UTI89 attached (b and c; thin arrows). Triple immunofluorescent staining (**d,e**) showed the urothelial apical staining of UPIa (far-red color; diamond-end arrows), surface-attached UTI89 (red; thin arrows) and nuclear translocation of RelA in urothelial umbrella cells (green; thick arrows). Scale bars: 200μm (**B**), 30 μm (**C**) and 50 μm (**D**).

To assess the *in vivo* effects of type 1-fimbriated uropathogenic *E. coli* (T1F-UPEC) on NF-κB pathways in urothelial cells, we first employed a transgenic reporter mouse line in which NF-κB responsive elements (NF-κB-RE) drive the expression of a β-galactosidase (*lacZ*) gene^46^,^47^,^48^ (Fig. 1A). We inoculated via the transurethral route 7–9 week old wild-type (Fig. 1B/a) and transgenic (Fig. 1B/b) female mice with UTI89, a T1F-UPEC strain originally isolated from a human patient with acute cystitis^31^. Twenty-four hours after inoculation, we assessed the β-galactosidase activity as an indication of NF-κB pathway activation, first using whole-mount bladders (Fig. 1B) and then frozen sections (Fig. 1C). While PBS-inoculated bladders had no discernible staining (Fig. 1B/a), those inoculated with UTI89 had numerous β-galactosidase-positive (blue) dots (Fig. 1B/b) indicating NF-κB activation. Frozen sections of UTI89-inoculated bladders of transgenic mice were then subject to β-galactosidase staining and adjacent (serial) sections were immuno-stained with an anti-*E. coli* antibody. β-galactosidase-positive cells were almost exclusively located at the superficial umbrella cell layer (Fig. 1C/a,C/b, black arrows). Anti-*E. coli* immunofluorescent staining of adjacent sections (Fig. 1C/a’, white arrows) showed excellent correlation between urothelial surface adhesion of the *E. coli* and positive β-galactosidase staining. These results strongly suggest that the adhesion of T1F-UPEC to the urothelial surface led to activation of NF-κB pathway in the urothelial umbrella cells.

We next examined whether the NF-κB activation triggered by T1F-UPEC in transgenic reporter mice could be reproduced with wild-type mice by immunofluorescent localization of RelA (p65), a major subunit of the canonical NF-κB pathway. RelA translocation from the cytoplasm to the nucleus is a hallmark of NF-κB activation^6^, but this had never been assessed in urothelium *in vivo* during UPEC infection. We observed that urothelial RelA in PBS-inoculated female mouse bladders was exclusively cytoplasmic, with the superficial umbrella cell layer exhibiting much weaker labeling than the basal and intermediate cell layers (Fig. 1D/a,D/a”). Inoculation of the mouse bladders with T1F-UPEC strain UTI89 resulted in prominent surface adhesion of the *E. coli* (Fig. 1D/b–e) as well as a marked increase of RelA in the nuclei as early as 1 hour post-inoculation. The RelA-positive nuclei were almost exclusively located in the urothelial umbrella cell layer (Fig. 1D/b–e). Bi-nucleated umbrella cells with both nuclei stained positively for RelA were also noted occasionally (Fig. 1D/c; right thick arrow; and Fig. 1D/e; lower-right thick arrows; also see later). A time-course analysis of urothelial NF-κB activation after UPEC inoculation indicated that RelA nuclear translocation was most prominent at 1 hour, much less so at 6 hours and almost undetectable at 12 and 24 hours (data not shown). In parallel experiments, we examined whether the non-canonical pathway was affected by T1F-UPEC with immunohistochemical staining using antibodies against RelB, p100/p52 and c-Rel. Neither cytoplasmic nor nuclear staining was observed in UPEC-infected urothelium, even though the antibodies detected strong signals in lymph nodes from mice with systemic bacterial infections (Supplemental Fig. 1). Taken together, our results establish that T1F-UPEC triggers a strong and rapid activation of the canonical NF-κB selectively in the umbrella cells of the urothelium.

### NF-κB Activation Is FimH-dependent

**Figure 2:**
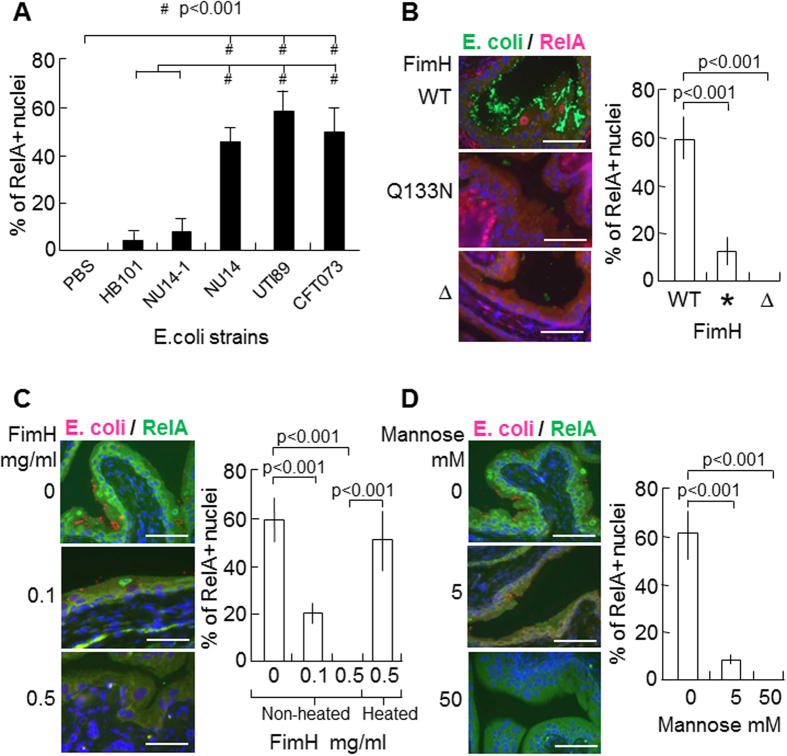
FimH-mediated adhesion of T1F-UPEC to urothelial surface was a prerequisite for NF-κB activation. (**A**) Effects of different *E. coli* strains on RelA translocation. Wild-type mice were transurethrally instilled with PBS or that containing the following bacteria: HB101 (a laboratory K12 strain not expressing the type 1 fimbriae); NU14-1 (a FimH deletion strain not expressing the type 1 fimbriae); NU14 (a clinical cystitis T1F-UPEC isolate which is the parental strain of NU14-1); UTI89; and CFT073 (a clinical pyelonephritis T1F-UPEC strain). Immunofluorescent staining was carried out on urinary bladders 1 h post-instillation and nuclei positively stained for RelA was counted and expressed as per 100 umbrella nuclei (see Materials and Methods for details). Note that UPEC strains expressing the type 1 fimbriae (NU14, UTI89 and CFT073), but not those non-type 1-fimbriated ones, induced significant nuclear translocation of RelA. (**B–D**) Effects of FimH-mediated adhesion of UPEC to urothelial cells on NF-κB activation. (**B**) Wild-type mice were transurethrally inoculated with the same number (10^8^ cfu) of T1F-UPEC strain UTI89, or a derivative of UTI89 expressing a FimH mutant (e.g., Q133N) (*) that lacks the ability to bind the terminal mannoses; or a derivative of UTI89 with FimH deletion (∆FimH) (∆). (**C–D**) Wild-type mice were transurethrally inoculated with the same number (10^8^ cfu) of T1F-UPEC strain UTI89 in the presence of 0, 0.1, 0.5 mg/ml of FimH or 0.5 mg/ml heat-inactivated (Heated) FimH (**C**), or in the presence of 0, 5 or 50 mM of D-mannose (**D**). One hour after inoculation, urinary bladders of all experimental groups (**B–D**) were processed for double-immunofluorescence staining using antibodies against *E. coli* and RelA. Representative images and semi-quantification of RelA-positive umbrella cell nuclei per 100 total umbrella nuclei were shown. Note that FimH mutation and deletion or addition of free FimH or D-mannose abolished UPEC-urothelial adhesion as well as NF-κB activation. Heat-inactivated FimH, however, failed to block NF-κB activation by UPEC (**C**). Scale bars: 100 μm (B–D).

To determine whether the canonical NF-κB activation in urothelial umbrella cells is a generalizable phenomenon, we tested additional *E. coli* strains. While a non-fimbriated laboratory strain HB101 and a FimH-deletion recombinant strain NU14-1 failed to elicit significant nuclear translocation of RelA (Fig. 2A), three independent T1F-UPEC clinical isolates (NU14, UTI89 and CFT073), at the same inoculum size (e.g., 10^8^ cfu in 25 μl), all induced marked RelA nuclear translocation at comparable levels. Of 100 umbrella cell nuclei counted, 50–60 were positive for nuclear RelA (Fig. 2A). These results further suggest that NF-κB in the urothelial cells at the steady state (e.g., prior to T1F-UPEC infection) is inactive and that the adherence of T1F-UPEC strains, but not the non-fimbriated ones, to the urothelial surface strongly activates the NF-κB pathway specifically in the urothelial umbrella cells.

To further study whether the adhesion of T1F-UPEC via its FimH adhesin to the urothelial surface was necessary for urothelial NF-κB activation, we engineered, from parental UTI89 strain, isogenic derivatives that harbored a point mutation in the FimH gene (UTI89/Q133N) (*) that abolished the mannose-binding ability of FimH or that harbored a deletion to disrupt FimH synthesis altogether (UTI89/∆FimH) (Fig. 2B). Compared with the wild-type strain, the FimH-defective strains led to a marked reduction of urothelial adhesion and a corresponding reduction of RelA-positive umbrella cell nuclei (parental UTI89 strain, ~60%; UTI89/Q133N (*) strain, ~10%; and UTI89/∆FimH strain, 0% (Fig. 2B). In addition, we found that (i) recombinant FimH^23^ inhibited the urothelial adherence of UTI89 as well as a corresponding reduction of RelA-positive umbrella cell nuclei in a dose-dependent fashion (~20% with 0.1 mg/ml FimH and 0% with 0.5 mg/ml FimH) (Fig. 2C); (ii) a large amount of FimH was not sufficient to activate NF-κB in urothelial umbrella cells (Fig. 2B, lower panel; also see Discussion); (iii) heat-inactivated FimH lost the ability to inhibit NF-κB activation by UTI89 (Fig. 2C); and (iv) the inclusion of D-mannose in the UTI89 inoculum also inhibited the urothelial adhesion of the bacteria and nuclear translocation of RelA in the umbrella cells (~5% with 5 mM D-mannose and 0% with 50 mM of D-mannose) (Fig. 2D). These data firmly established that urothelial adhesion of T1F-UPEC via FimH was critical for NF-κB activation.

### Persistent Type 1-fimbrial Expression Expands and Prolongs NF-κB Activation in Urothelial Cell Layers

**Figure 3:**
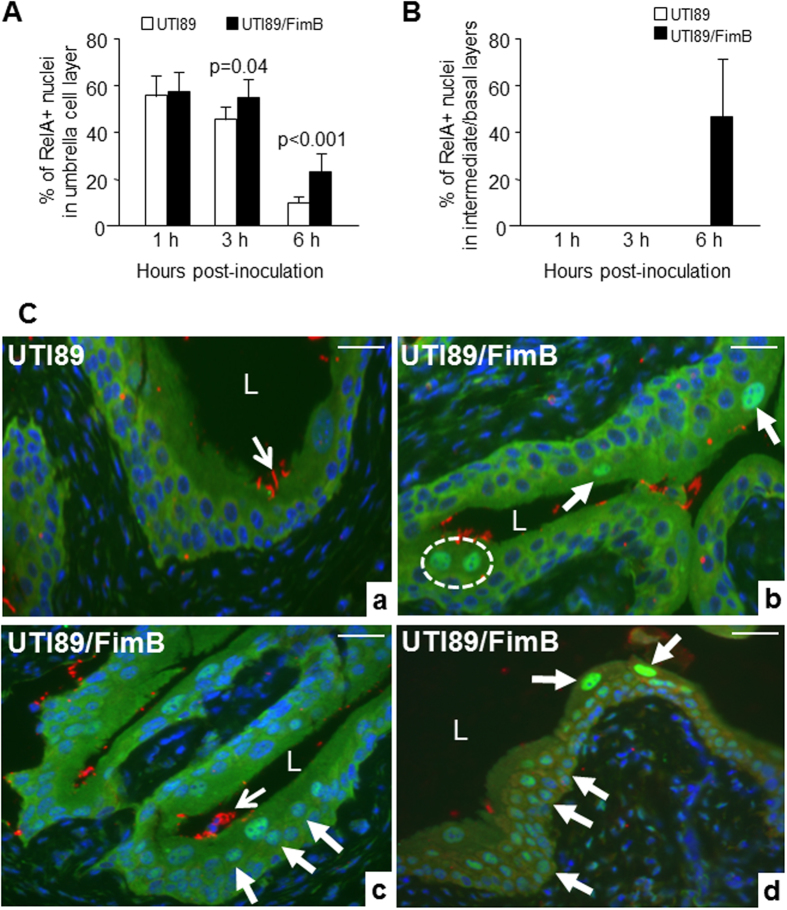
Persistent type 1 fimbrial expression of UPEC led to NF-κB activation in lower urothelial cell layers. Parental UTI89 UPEC strain (10^8^ cfu) and its derivative over-expressing a plasmid-encoded FimB (10^8^ cfu) that “locks on” the expression of type 1 fimbriae were transurethrally inoculated into wild-type mouse bladders and the urothelial sections were subject to anti-RelA staining 1, 3 and 6 h post-inoculation. Note that the persistent type 1 fimbrial expression in UTI89/FimB prolonged NF-κB activation (e.g., from 1 h to 3 h and 6 h post-inoculation) in umbrella cells ((**A**) and (**C**)/b-d), and induced NF-κB activation even in intermediate (thick arrows in C/c) and basal cells (C/d) 6 h post-inoculation. Dashed circle in C/b marks a potentially bi-nucleated umbrella cell with both nuclei being positive for RelA. Thin arrows point to *E. coli*; L denotes bladder lumen. Scale bars: 50 μm (**C**).

Because type 1-fimbriation is controlled by “on” and “off” phase variation, particularly *in vivo*, we tested whether persistent type 1-fimbrial expression could affect the extent of urothelial NF-κB activation. To do so, we transfected the type 1-fimbriated UTI89 strain with a plasmid encoding FimB, a recombinase of the type 1 fimbrial operon that keeps transcription of the type 1-fimbrial genes in an “on” position^49^. While at 1 h post-inoculation this strain and its parent wild-type strain had a similar level of NF-κB activation in the umbrella cells, at 3 h and 6 h the FimH-on strain caused NF-κB activation in significantly greater numbers of umbrella cells (Fig. 3A–C). Moreover, infection with this FimH-on strain triggered NF-κB activation not only in umbrella cells, but also in the intermediate and basal cells at 6 h (Fig. 3B,C/c-d), a phenomenon that happened rarely with any wild-type, T1F-UPEC strains.

### NF-κB Is Activated in Multiple Nuclei in the Multinucleated Umbrella Cells

**Figure 4:**
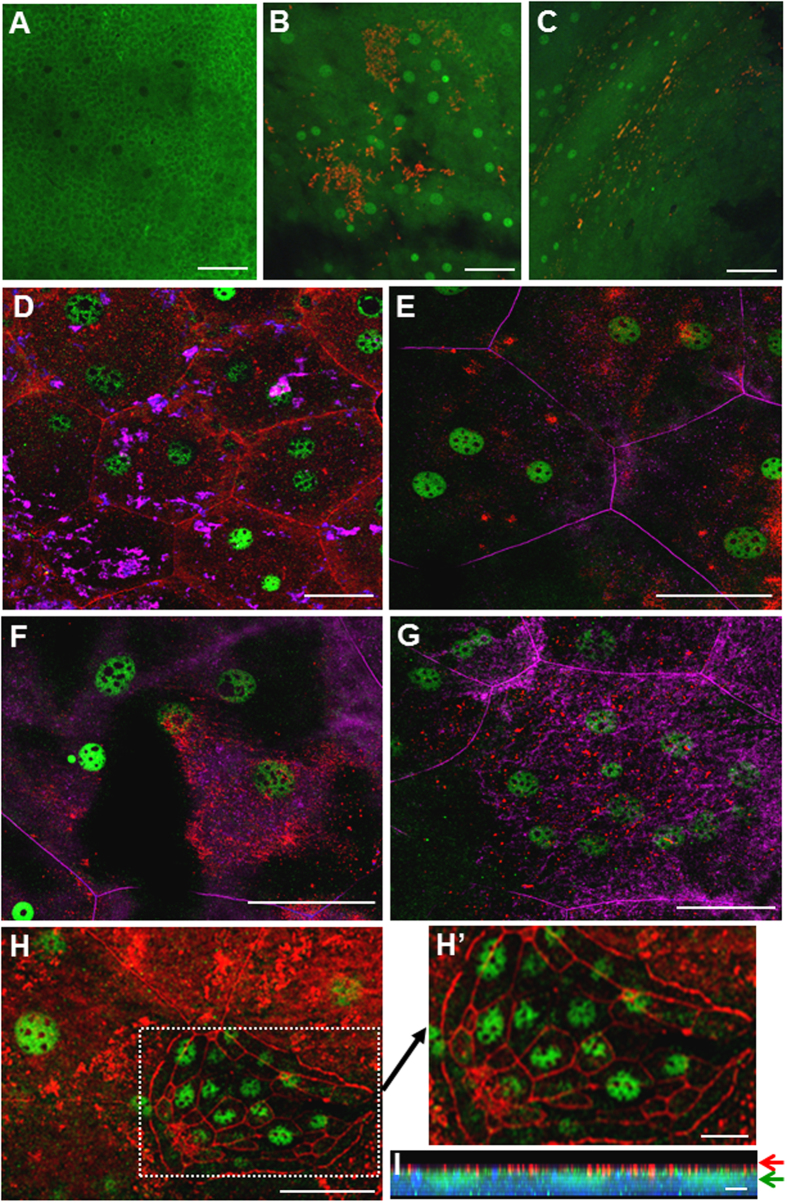
Multiplicity of NF-κB activation in multinucleated umbrella cells. Wild-type mouse bladders were transurethrally inoculated with either PBS (**A**) or UPEC strain UTI89 (10^8^ cfu; (**B–I**)) and 3 h post-inoculation the bladders were excised, fixed in 4% paraformaldehyde, permeabilized in 0.5% Triton. The whole mount preparation was stained with different combinations of antibodies against *E. coli*, NF-κB, ZO-1 and was viewed with confocal fluorescent microscopy. (**A**) Exclusively cytoplasmic localization of NF-κB in a PBS-inoculated bladder. (**B,C**) Nuclear translocation of NF-κB (green, round dots) with surface-adherent UPEC (red) in UTI89-inoculated bladders. (**D–G**) Marking of umbrella cell borders with anti-ZO-1 (red in (**D,H** and **H’**) and pink in (**E–G**)) revealing NF-κB activation in multiple nuclei (2-11 nuclei) in umbrella cells. Anti-*E. coli* staining showed surface attachment (pink in (**D**) and red in (**E–G**)). (**H** and **H’**) Detachment of an umbrella cell (inside the dash box) exposing multiple differentiating intermediate cells each surrounded by ZO-1 that exhibited RelA nuclear translocation. (**I**) Z-stacked images of an area in (**E**) showing surface-adherent UPEC (red) and nuclei-translocated RelA (green). Scale bars: 200 μm (**A–C**), 25 μm (**D–H**), 10 μm (**H’**) and 4 μμm (**I**).

As mentioned, we found that, in some cross-sections, both nuclei of the binucleated umbrella cells seemed to undergo NF-κB activation by T1F-UPEC (Fig. 1C/b,1D/c,1D/e and 3C/b – dashed circle). We examined this issue more closely using whole mount mouse bladders and confocal immunofluorescent microscopy to visualize the T1F-UPEC, RelA and the tight junction component ZO1 (for the umbrella cell boundary). The results showed even more clearly that RelA was cytoplasmic in PBS-inoculated bladders (Fig. 4A) and that it became nuclear upon UTI89 infection (Fig. 4B,C) in cells with surface-attached *E. coli*. While approximately 80% of the umbrella cells contained two nuclei (Fig. 4D), the remainder had more (e.g., 3 as shown in Fig. 4E; 5 in Fig. 4F and 11 in Fig. 4G). Strikingly, NF-κB was activated in all the nuclei within a given umbrella cell (Fig. 4D–G). Occasionally, when an umbrella cell was sloughed off exposing intermediate cells that started to differentiate and express ZO-1, NF-κB activation was observed even in the single-nucleated intermediate cells (Fig. 4H,H’). Z-stack of the confocal images confirmed the association between surface attachment of *E. coli* and nuclear translocation of NF-κB (Fig. 4I).

### Urothelial Nuclear NF-κB Extracts from UPEC-infected Bladders Are Capable of Binding to Target-DNA and Inducing Pro-inflammatory Cytokines

To determine whether nuclear NF-κB in the urothelial umbrella cells upon T1F-UPEC infection was functionally active and capable of binding target DNA and transcribing target genes, we extracted nuclear proteins of the urothelial cells from PBS- and T1F-UPEC (UTI89)-inoculated bladders, incubated them with immobilized NF-κB-binding consensus DNA, followed by detection of bound RelA by ELISA. T1F-UPEC-exposed urothelial cells showed a 250% increase in RelA that bound to the target DNA over PBS-exposed urothelial cells (Supplemental Fig. 2A). Because increased target-sequence binding of NF-κB may lead to increased transcription of NF-κB target genes, particularly those encoding the pro-inflammatory cytokines, we assessed the urothelial and urinary levels of TNFα, IL1α, IL1β and IL12 by ELISA in PBS- and UTI89-inoculated mice. We detected a significant increase of TNFα in both urothelial and urine samples as early as 1 h post-inoculation (Supplemental Fig. 2B/a,B/b), most likely due to an acute-phase response of the urothelium. This increase leveled off at 6 and 16 h post-inoculation. The induction of IL1α and IL1β lagged behind TNFα with a significant rise in urothelium and urine at 6 h. While the urinary IL1α decreased at 16 h (Supplemental Fig. 2B/d), urothelial IL1α and IL12 increased at 16 h (Supplemental Fig. 2B/c,B/g), probably due to a secondary production of these cytokines by inflammatory cells that had infiltrated the urothelium. The increase of target DNA binding and production/secretion of urothelial pro-inflammatory cytokines upon T1F-UPEC infection further suggest, on a functional level, that NF-κB is activated by T1F-UPEC in urothelial cells.

### Toll-like Receptor 4 Is Required for Urothelial NF-κB Activation

**Figure 5:**
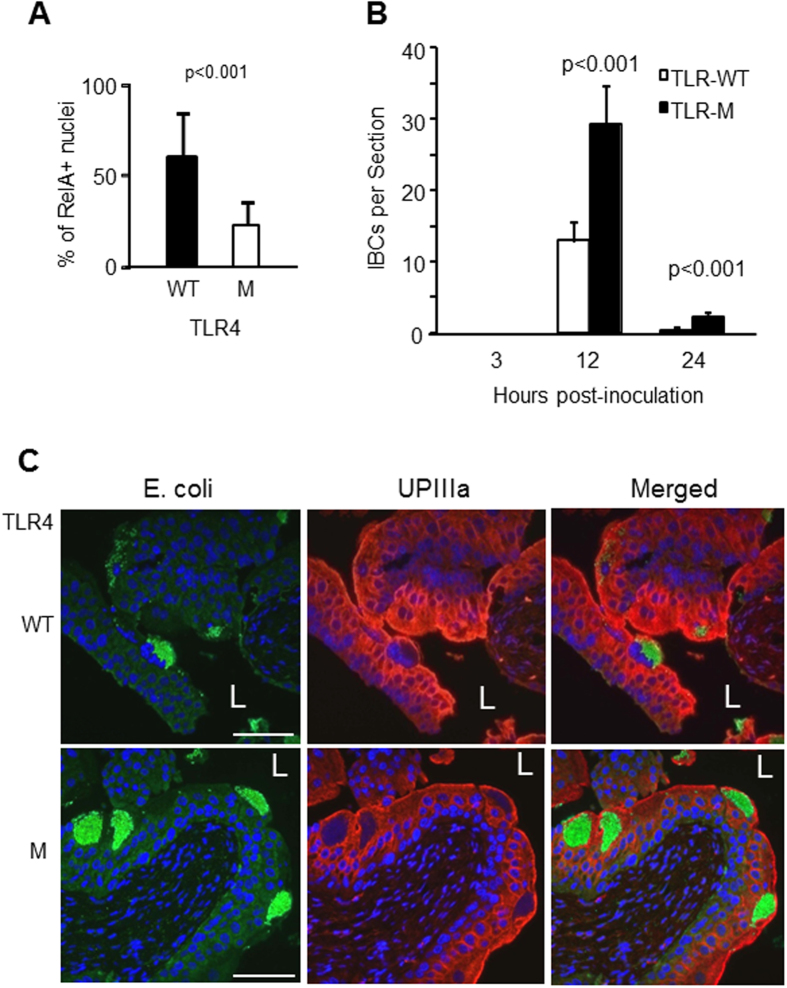
Toll-like receptor 4 mediated T1F-UPEC-dependent NF-κB activation. (**A**) Wild-type and TLR4-mutant mice were transurethrally instilled with T1F-UPEC strain UTI89 and their urothelia were stained with anti-RelA antibody. Note a significantly smaller number of RelA-positive umbrella cell nuclei in TLR-4 mutant mice than in the wild-type control (n = 3; p < 0.001). (**B,C**) Effects of TLR4/NF-κB deficiency on persistence of intracellular T1F-UPEC. Wild-type and TLR4 mutant mice were infected via the transurethral route with T1F-UPEC strain UTI89. Urinary bladders were procured at 3, 12 and 24-hours post-infection, paraffin-sectioned and double-immunofluorescence-stained with anti-*E. coli* and anti-uroplakin IIIa antibodies and counter-stained with DAPI. (**B**) Enumeration of intracellular bacterial communities (IBCs) at different time points (n = 3 mice/genotype/time point; p < 0.001 between mutant and wild-type mice at 12 and 24 hours). IBC number was expressed as per cross-section (see Materials and Methods for details). (**C**) Representative images showing greater number of and larger IBCs in umbrella cell layer of the TLR4 mutant mice than that in wild-type controls. Scale bars: 100 μm (**C**).

Since Toll-like receptor 4 (TLR4) is a specific receptor for lipopolysaccharide (LPS)^35^, a major outer membrane component of T1F-UPEC, and is known to be expressed by the urothelial cells^50^, we tested whether it played a role in mediating NF-κB activation by T1F-UPEC. Inoculation of wild-type (C3H/HeOuJ) female mouse bladders with LPS indeed led to marked nuclear translocation of RelA (Fig. 5A). In comparison, the C3H/HeJ mice, which differed from C3H/HeOuJ control mice in harboring an inactivating mutation in TLR4^51^,^52^, had a three-fold fewer RelA-positive umbrella cell nuclei (20% in mutant mice vs. 60% in wild-type mice; p < 0.001) (Fig. 5A), but two to three fold more intracellular bacterial communities (IBCs) (mutant:~30 and 3 IBCs/section at 12 and 24 hours post-inoculation, respectively; wild-type: ~12 and ~1 IBC/section at 12 and 24 hours, respectively) (Fig. 5B,C). Together, these data strongly suggest that higher level of NF-κB activation can reduce bacterial persistence in the bladder, and that TLR4 plays a critical role in mediating NF-κB activation by T1F-UPEC (see later).

### Chemical Inhibition of NF-κB Results in Bacterial Persistence in the Bladder

**Figure 6:**
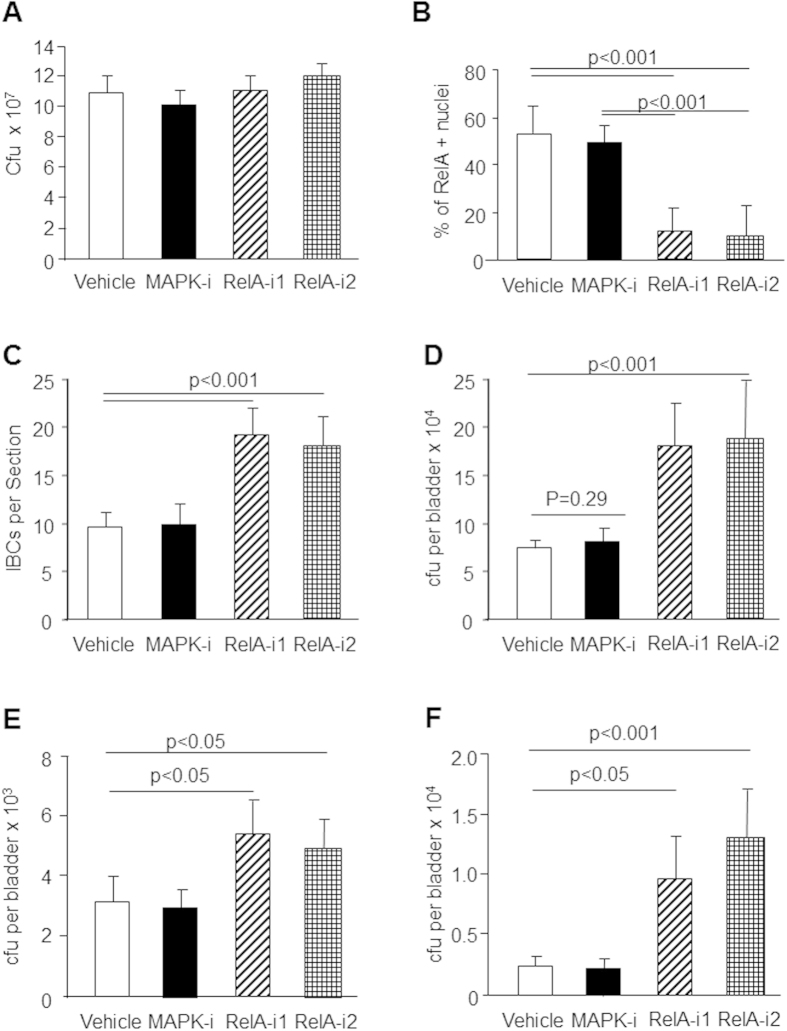
Inhibition of NF-κB pathway led to increased numbers and delayed clearance of intracellular bacterial communities formed by T1F-UPEC. (**A**) Culture of UTI89 *in vitro* for 1 hour in the presence of vehicle (DMSO), MAPK inhibitor (MAPK-i), RelA inhibitor 1 (RelA-i1) and 2 (RelA-i2) showing that these inhibitors had no significant effects on bacterial growth. (**B**) RelA antibody staining carried out on bladders 1 h after UTI89 inoculation showing marked reduction of positive umbrella cell nuclei with the two RelA inhibitors (n = 3). (**C**) Enumeration of intracellular bacterial communities (IBCs) on mouse bladders at 12 hour post-inoculation showing greater number of IBCs in the two RelA-i groups than in the controls. (**D**) Bacterial culture of mouse bladders at 24 hour post-inoculation showing great number *E. coli* in the two RelA-i groups than in the controls (5 mice per group). (**E,F**) Bacterial culture of mouse bladders at 3 days and 7 days post-inoculation, respectively, showing significantly great number of *E. coli* in the two RelA-i groups than in the controls (5 mice per group).

To examine whether NF-κB status could affect the various stages of UTI pathogenesis, we included chemical inhibitors of NF-κB in the UTI89 inocula. When tested *in vitro* on LB-agar plates, neither the solvent nor the three chemical inhibitors affected bacterial growth (Fig. 6A). Significant differences were observed, however, when the different inocula were introduced into the wild-type mouse bladders *in vivo* (Fig. 6B–D). Both of the RelA inhibitors, but not the solvent or MAPK inhibitor, caused a marked reduction of RelA-positive umbrella cell nuclei at 1 h post-inoculation of UTI89 (Fig. 6B), a significant increase of IBCs at 12 h post-inoculation (Fig. 6C), as well as a significant increase of bacterial counts 24 h post-inoculation (Fig. 6D). Prolonged retention of UTI89 was also observed in bladders analyzed at 3 days and 7 days post-inoculation (Fig. 6E,F). These data indicate that inhibition of the NF-κB pathway resulted in a significant delay in UPEC clearance from the infected bladders.

## Discussion

### Type 1-fimbriated Uropathogenic *E. coli* Strongly Activates, rather than Inhibits, NF-κB of Urothelial Cells *In vivo*

Although exactly how the NF-κB pathway of the urothelial cells responds to UPEC is of clear importance to elucidating the pathogenesis of urinary tract infections, existing data based primarily on cultured cells are controversial^31^,^32^,^33^,^34^,^35^,^36^,^43^. Using mouse models of ascending urinary tract infection, we provide multiple lines of evidence demonstrating that (i) type 1-fimbriated uropathogenic *E. coli* (T1F-UPEC) triggers the trans-activation of NF-κB-responsive elements in transgenic reporter mice (Fig. 1A–C); (ii) T1F-UPEC induces rapid nuclear translocation of the canonical NF-κB component RelA, without affecting the non-canonical NF-κB components, in urothelial cells of the wild-type mice (Fig. 1D; Supplemental Fig. 1); (iii) the nuclear fraction of NF-κB from UPEC-challenged urothelial cells binds to NF-κB-responsive DNA and trans-activates pro-inflammatory cytokine TNF-α, IL1α and IL1β genes (Supplemental Fig. 2); and (iv) the NF-κB activation in urothelial cells is highly reproducible with divergent clinical strains of T1F-UPEC but not with non-type 1-fimbriated laboratory controls (Fig. 2A). Our data, based on physiologically relevant *in vivo* mouse models, establish for the first time that the canonical NF-κB pathway in urothelial cells is strongly activated, instead of being inhibited as reported previously, by T1F-UPEC. Our findings have important implications on urothelial biology and pathogenesis of urinary tract infections as discussed below.

### Urothelial NF-κB Activation Requires a Dual Ligand/receptor System

**Figure 7:**
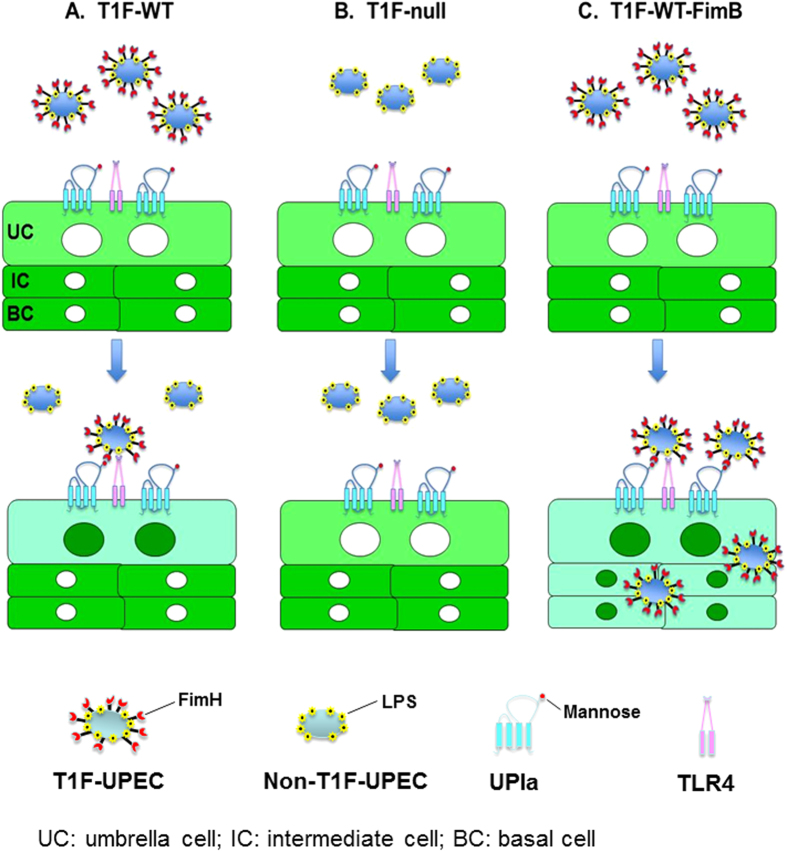
Roles of dual ligand-receptor systems (FimH/UPIa receptor and LPS/TLR4 receptor) in NF-κB activation and UPEC invasion of the host urothelial umbrella cells: a model. (**A**) With a steady-state expression of type 1 fimbria in wild-type UPEC (T1F-WT; filled blue oval with filaments capped with FimH adhesin), FimH of the UPEC bind to the high-mannose moiety of UPIa and in the meantime bring LPS to interact with TLR4 on the urothelial surface, thus triggering NF-κB nuclear translocation. However, since type 1 fimbriation may be transient *in vivo* due to phase variation, NF-κB activation occurs only in the umbrella cell layer (filled green cycles). (**B**) In the absence of type 1 fimbria (T1F-null; filled blue oval), UPEC are incapable of binding to the mannose-bearing uroplakin (UP) Ia receptor of the urothelial surface and hence unable to bring LPS (yellow flowers on the bacterial wall) close to the TLR4 on the urothelial surface to activate NF-κB. Thus, NF-κB (green) remains exclusively cytoplasmic with no nuclear translocation (open circle) in all urothelial layers. (**C**) Persistent expression of type 1 fimbria in UPEC (as achieved by FimB overexpression – T1F-FimB) enhances urothelial surface binding and invasion into deeper urothelium and induces nuclear translocation of NF-κB not only in umbrella cells but also in intermediate and basal cells. This model underscores the critical importance of type 1 fimbria-mediated adhesion of UPEC to the urothelial cells and the dual ligand/receptor systems (e.g., FimH/UPIa and LPS/TLR4) as prerequisites for activating the canonical NF-κB pathway of the urothelium.

A key observation we made in this study is that NF-κB activation in urothelial cells is dependent on the adhesion of uropathogenic *E. coli* to the urothelial surface. First, clinical *E. coli* strains from patients with acute urinary tract infections, including UTI89, NU14 and CFT073, that express the type 1-fimbriae and bind avidly to the urothelial surface, strongly activate NF-κB. Second, urothelial surface attachment of these clinical strains shows an excellent correlation with NF-κB activation in both transgenic reporter mice (Fig. 1A) and wild-type mice, on both cross-sections (Fig. 1D) and whole mount bladder preparations (Fig. 4). Third, isogenic derivatives of these clinical strains that only differ from their parental counterparts in lacking a functional FimH (e.g., NU14-1, UTI89/∆FimH) or acquiring a loss-of-function FimH mutation (e.g., UTI89/Q133N-FimH) lose the ability to bind to the urothelial surface and fail to activate NF-κB (Fig. 2B). Finally, blocking the binding of T1F-UPEC to urothelial surface by free FimH or D-mannose also blocks NF-κB activation (Fig. 2C,D). It is clear, therefore, that the adhesion of T1F-UPEC via its FimH lectin to the unmodified mannose of uroplakin Ia receptor of the urothelial surface is a prerequisite for T1F-UPEC to activate NF-κB (Fig. 7). We believe that this FimH:uroplakin receptor interaction is crucially important, because it brings the pathogen-associated molecular patterns (PAMPs) on T1F-UPEC, particularly lipopolysaccharide (LPS), to the close proximity of pattern recognition receptors (PRRs) such as TLR4^53^,^54^ on the urothelial surface. Such a close physical proximity and high local LPS concentration allow the LPS to interact favorably with TLR4 and trigger a signaling cascade that activates the NF-κB (see model Fig. 7A). While isogenic strains with FimH-deletion or -mutation (e.g., NU14-1, UTI89/∆FimH, UTI89/Q133N-FimH) carry the same type and amount of PAMPs (e.g., LPS), they lack the ability to bind to the urothelial surface and as a result are incapable of bringing a high concentration of PAMPs to urothelial surface to activate NF-κB (Fig. 7B). Urothelial NF-κB activation therefore appears to require a dual ligand/receptor system, one involving FimH/uroplakin Ia and another involving LPS/TLR4 (Fig. 7A and see below). By demonstrating a major requirement of the dual ligand/receptor system in urothelial NF-κB activation, we are by no means ruling out the involvement of other bacterial and/or host factors in influencing the outcome of UPEC infections. Further studies are clearly needed to define host urothelial responses to specific infectious conditions.

### Type 1 Fimbriation Affects Both UPEC Pathogenicity and Host Defense

The type 1 fimbria as a major virulence factor for UPEC to cause urinary tract infection has been well established^29^,^30^. Not only are the fimbriae important for the bacteria to latch onto the urothelial surface thus avoiding being washed away during micturition, they are also critical for the bacteria to invade into the cytoplasm of the superficial urothelial cells. Because the intracellular UPEC are protected from the antibiotics and host immune cell attacks, they serve as a likely resource of recurrent infections^29^,^30^. Contrary to the role of type 1 fimbriae as a major virulence factor for UPEC, our present study suggests an opposite role for the fimbriae in eliciting innate host defenses because they are indispensable for activating the NF-κB pathway and stimulating pro-inflammatory cytokines (Figs 1, 2A and 4; Supplemental Fig. 2). These host responses are antagonistic to the UPEC infection as they help to reduce or clear UPEC from the infected urothelium. The type 1 fimbriation could on the one hand enhance bacterial virulence, and on the other hand, activate host innate defenses that limit the bacterial infection.

### Multinucleated Umbrella Cells Act as the Primary Initiator of Innate Defenses of Mammalian Urothelia

Another key observation of our present work is the highly selective nature of NF-κB activation in the urothelial umbrella cells upon bladder infection by T1F-UPEC (Figs 1,2 and 4). This occurs despite the fact that, at the steady state, the umbrella cells contain a relatively low level of cytoplasmic NF-κB/RelA, compared with the intermediate and basal cells (Fig. 1D/a). Within one-hour of transurethral inoculation of wild-type T1F-UPEC, almost all cytoplasmic NF-κB/RelA of the umbrella cells, but little if any of the cytoplasmic NF-κB/RelA of the intermediate and basal layers, has translocated into the nucleus (Fig. 1D). Such a rapid and umbrella cell-specific initial response of NF-κB is likely due to the direct interaction of T1F-UPEC with the apical surface of the umbrella cells^22^,^23^,^24^,^25^,^45^. The lack of such direct interactions for the intermediate and basal cells may explain the preferential activation of the umbrella cells (Figs 1,2 and 4). However, this boundary can be broken when the type 1 fimbriae are more persistently expressed in T1F-UPEC strain UTI89 over-expressing the FimB which locks on the type 1 fimbrial expression. In this case, NF-κB activation could be observed even in the intermediate and basal cells (Fig. 3). Consistent with this, NF-κB activation in intermediate cells also occurs when the overlying umbrella cells are detached thus breaching the barrier (Fig. 5H,H’). These data indicate that, although umbrella cells are clearly the frontline in urothelial defenses against the T1F-UPEC, the lower cell layers possess the necessary signaling cascade for NF-κB activation and can be involved, perhaps in a “backup” role, in the inflammatory responses at a later infection stage and/or during the “on-phase” of the type 1 fimbriae (Fig. 7C). Thus, our data suggest a new concept that phase variation of T1F-UPEC may affect the extent, duration and cell layer involvement of NF-κB activation (see below).

Our finding that NF-κB is activated in all the nuclei within a single umbrella cell is also unexpected. While bi-nucleation of a fraction of urothelial umbrella cells was noted previously and believed to be a result of either failed cytokinesis of the umbrella cells or fusion of the intermediate cells, thus far not a single functional attribute has been assigned to umbrella cell bi-nucleation^9^. By confocal immunofluorescent microscopic imaging of the whole mount mouse bladders in conjunction with tight junction markers, we found that almost all the urothelial umbrella cells are multinucleated with about 80% containing 2 nuclei, 15% containing 3–5 and the remaining containing more than 5 (up to 11) (Fig. 4). Remarkably, NF-κB activation by T1F-UPEC occurs in all the nuclei within an umbrella cell. Activation of NF-κB activation in multiple nuclei may be functionally important as it can amplify the amounts of messenger RNAs encoding pro-inflammatory cytokines and chemokines more than would a single nucleus.

Multinucleated cells are found in other tissue/organ types such as skeletal and cardiac muscle, placenta, liver, brain and blood cells^55^. Limited evidence suggests that multinucleation contributes to development and differentiation. While umbrella cells are undoubtedly a hallmark of terminal differentiation of the urothelium, our current data suggest yet another important function of these multinucleated umbrella cells in pro-inflammatory signal amplification in urothelial defense against T1F-UPEC.

### Urothelial NF-κB Activation *In vivo* by T1F-UPEC Is Dependent on TLR4 Signaling

Like other mucosal epithelia, urothelium is believed to express pathogen recognition receptors, of which the TLR family has received most attention. Although both RT-PCR and immunohistochemistry show the expression of TLR-2 to −4, −5, −7 and −9 in human urothelium^50^ and TLR-11 in mouse urothelium^56^, the immunohistochemical staining of some of the TLRs (e.g., 2, 3, 5 and 7) is somewhat equivocal^50^. To date, none of these TLRs has been functionally linked to the NF-κB status in the urothelium *in vivo*. Our data showed that mutant mice with functionally defective TLR4 had significantly fewer RelA-positive umbrella cell nuclei than the isogenic control mice with functionally intact TLR4 (Fig. 5B). These results strongly suggest TLR4 as the major PRR in the UPEC-triggered NF-κB activation in the urothelial umbrella cells. While several recent studies reveal that FimH can also serve as a TLR4 ligand in professional phagocytes^57^,^58^,^59^, we show here that the binding of FimH to the umbrella cell surface is necessary but insufficient to activate the TLR4/NF-κB pathway (Fig. 2C). In fact, a high concentration of FimH (e.g., 0.5 mg/ml) not only did not activate the umbrella cell NF-κB, but completely blocked UPEC from activating the NF-κB (Fig. 2C). The mere binding of T1F-UPEC to the urothelial surface also could not trigger NF-κB activation in the absence of a functional (e.g., TLR4) signaling pathway because, in the TLR4 mutant mice where NF-κB activation is compromised, the amount of uroplakins (Fig. 5C) and the initial urothelial adhesion of UPEC (data not shown) were not lower than those in the wild-type controls. Our data from the TLR4 mutant mice lend further support to our suggestion that urothelial NF-κB activation relies on a dual ligand/receptor system (e.g., FimH/UPIa and LPS/TLR4; Fig. 7).

### Functional Status of Urothelial NF-κB May Affect Clinical Presentation of Urinary Tract Infections

Our suggestion that NF-κB activation serves as an important innate defense of the urothelium against T1F-UPEC is further supported by the fact that, when this pathway is inhibited, the mouse urothelium is much less efficient in clearing the intracellular bacterial communities. In the TLR4 mutant mice where urothelial NF-κB activation is severely compromised, a much greater number of IBCs were present at both 12- and 24-hour post-inoculation in the TLR4 mutant mice than in the wild-type controls (Figs 5B,C). Inclusion of two different NF-κB pathway inhibitors, but not a MAPK inhibitor, in the UPEC inocula also delayed IBC clearance from the infected wild-type mouse bladders and prolonged UPEC colonization (Fig. 6) in a manner strikingly similar to that of the TLR4 mutant mice. These data indicate that the functional status of NF-κB in the urothelium has a direct effect on the time course of urinary tract infections.

While the NF-κB status of the urothelium can have a clear impact on the pathogenesis of acute cystitis, it remains to be seen whether it influences the disease course and clinical presentation of other forms of microbe-related conditions of the urinary system, including asymptomatic bacteriuria (ABU)^60^, recurrent urinary tract infections and pyelonephritis. Svanborg and colleagues found a strong correlation between low levels of TLR4 expression due to single nucleotide polymorphisms in the TLR4 promoter and the existence of ABU^61^,^62^. Because TLR4 acts upstream of NF-κB, it is possible that reduced NF-κB signaling, hence reduced inflammatory responses to the ABU strains, might play a part in the absence of overt symptoms in some of these patients. On the other hand, if the NF-κB signaling is too severely compromised to the point that urothelial cells can no longer clear the pathogens, the host might develop recurrent infections or even upper tract infections. In this regard, the varied levels of NF-κB activation of the host urothelial cells in response to urinary pathogens may affect the severity, disease course and presentation of the various forms of urinary tract infections. Finally, recent studies from independent groups showed that the healthy urinary tract may not be entirely sterile as it may contain microbes that are not cultivatable with conventional methods^63^,^64^. If this is indeed the case, the urothelial cells may act much like the intestinal counterpart in being normally exposed to a population of commensal microbes. It would be of tremendous interest and importance to know whether normal urothelial homeostasis depends on these commensal microbes and how the urothelial cells distinguish the commensal microbes from the pathogens by differentially regulating the NF-κB pathway. The urinary tract can potentially be another useful model system for studying the symbiotic relationships between commensal microbes and mucosal epithelial cells.

## Methods

### Experimental Animals

Transgenic reporter mice harboring a transgene consisting of NF-κB responsive elements upstream of a minimal promoter and a *lacZ* reporter fused in-frame at the 5′-end to a nuclear localization sequence (NF-κB-RE/NLS-*lacZ*)^46^,^47^,^48^ was re-derived into and continuously maintained in the C57BL/6 inbred background in a specific-pathogen-free facility. Genotyping was performed with PCR using *lacZ*-specific primers and genomic DNA extracted from biopsied tails. Wild-type mice in 129/SvEv in-bred background were purchased from Taconic (Germantown, NY). Toll-like receptor 4 (TLR4) mutant mice C3H/HeJ (Tlr4^LPS-d^) and TLR4-wild type control mice C3H/HeOuJ (Tlr4^LPS-n^) were purchased from the Jackson Laboratory (Bar Harbor, Maine). All experiments on animals were performed in accordance with federal and local regulations and after official approval from the Institutional Animal Care and Use Committee of New York University School of Medicine.

### Bacterial Strains and Culture

The following *E. coli* strains were used: (1) UTI89, a type 1-fimbriated uropathogenic *E. coli* (T1F-UPEC) isolate from an acute cystitis patient^31^; (2) UTI89/Q133N, a recombinant strain engineered from the parental UTI89 strain with a point mutation converting codon 133 of FimH adhesin from Q to N, thus abolishing FimH’s ability to bind terminal mannoses; (3) UTI89/∆FimH, a recombinant strain engineered from the parental UTI89 strain with FimH adhesin deleted; (4) CFT073 (from ATCC), a T1F-UPEC strain isolated from the blood and urine of a patient with acute pyelonephritis^65^; (5) NU14, a T1F-UPEC strain isolated from an acute cystitis patient^66^; (6) NU14-1, a recombinant strain derived from NU14 with FimH adhesin deleted^67^; (7) HB101, a non-fimbriated, K12 laboratory *E. coli* strain (ATCC)^68^; and (8) UTI89/FimB, a derivative strain from UTI89 after transfection with a plasmid encoding the FimB gene (pACYC177/FimB), which “locks on” the type 1 fimbrial expression^49^.

All the *E. coli* strains were prepared under the identical culture condition. Briefly, frozen glycerol stocks were used to streak the LB agar plates and single colonies were grown in LB broth at 37 °C statically for 3 consecutive passages (one passage/24 hours) to facilitate fimbriation which was verified by incubation with 1% Baker’s yeast suspended in PBS. Bacteria from the last culture were washed twice by centrifugation and resuspension in PBS and only freshly cultured bacteria were used for experimental bladder infection.

In assessing the extent of bladder infections, bladders were aseptically removed, washed using sterile PBS and then homogenized in 0.05% Triton X-100 in PBS and serially diluted and plated on LB agar plates for enumeration of colony forming units (CFU).

### Experimental Bladder Infection

Seven to nine week-old female mice were anesthetized by intramuscular injection of ketamine (100 mg/kg) and xylazine (10 mg/kg). Twenty-five microliters of 10^8^ cfu of various *E. coli* strains were resuspended in PBS and inoculated via the transurethral route into the mouse bladders via a catheter designed for mice (inner diameter 0.28 mm)^69^. The same volume of PBS without bacteria was instilled via the transurethral route routinely as a negative control. In addition, in testing the importance of the adhesion of T1F-UPEC to urothelial surface on NF-κB activation, various *E. coli* strains were preincubated with purified lectin domain of FimH or various concentrations of D-mannose as described in the figure legends. The mixtures were then inoculated into the mouse bladders. Inoculated mice were sacrificed at different time points and their urinary bladders procured for analyses.

### β-galactosidase Assay

For *in situ* β-galactosidase detection on whole mount bladders, 24 hours after the female NF-κB-RE/NLS-*lacZ* transgenic mice were inoculated with PBS or T1F-UPEC strain UTI89, their urinary bladders were removed and incubated at 37 °C for 20 h in X-gal solution containing 4 mM potassium fericyanide, 4 mM potassium ferrocyanide, 2 mM magnesium chloride, 1 mg/ml X-gal in PBS (pH 7.4). The bladders were then placed on a glass slide and covered with a cover slip to flatten the wall and photographed on the mucosa and serosa sides. For β-galactosidase detection in tissue-sections, 5-μm frozen sections were cut on a Cryostat, post-fixed in 2% formaldehyde and 0.2% glutaraldehyde in PBS (pH 7.4) for 10 min at room temperature and then incubated for 2 h in the X-gal solution described above.

### NF-κB Activity Assay

Urothelial nuclear proteins were obtained using a Nuclear Extraction kit (Signosis, Sunnyvale, CA) and used for measuring NF-κB activity using a kit (Signosis, Sunnyvale, CA). Briefly, nuclear proteins were incubated with pre-coated NF-κB consensus sequence oligo-plate for 30 min at room temperature. After washing, the bound NF-κB was detected with an antibody against RelA and a secondary antibody conjugated with horseradish peroxidase. The colorimetric substrate was then added and optical density determined in a microplate reader at 450 nm.

### Cytokine Measurement

For determination of urine cytokines, spot urine samples were collected at the time of mouse sacrifice (n = 3 mice/time point). For measurement of urothelial cytokines, urothelial cells were scraped off using a drug spatula from the inner surfaces of the mouse bladders into PBS at different time points of stoppage of bladder infection. Urothelial cells from 4 identically treated mice were combined, spun down (800 x g) and re-suspended in 150 μl extraction buffer containing 20 mM Hepes (pH 7.5), 150 mM NaCl, 1 mM EGTA, 1.5 mM MgCl2, 10% glycerol, 1% Triton X-100 plus a protein inhibitor cocktail (Thermo Fisher Scientific). Three combined samples were used per time point. Fifty microliters of urine or 150 μg of total urothelial proteins as measured by BCA reagent (Thermo Fisher Scientific) were used for ELISA using commercial kits specific for mouse TNFα, IL1α, IL1β and IL12 (Thermo Fisher Scientific).

### Immunofluorescence Staining of Bladder Cross-sections

Mice (n = 3 per T1F-UPEC strain) were infected and sacrificed at different time points. Their bladders were removed, processed and embedded routinely in paraffin. Four-μm thick sections were cut along the midline of bladder neck to dome. One section was chosen from every 10 serial sections and a total of 10 sections were used for immunofluorescence staining. Briefly, the deparaffinized sections were subject to non-specific site blocking in 5% BSA in PBS for 1 h at room temperature followed by incubation with a primary antibody overnight at 4^0^C. The primary antibodies, their manufacturers and dilutions were: rabbit or goat anti-*E. coli* (Maine Biotechnology Service; 1:300); mouse anti-RelA (Santa Cruz Biotechnology; 1:200); rabbit anti-RelA (Abcam; 1:2,000); mouse anti-UPIIIa (70; 1:300); rabbit anti-UPIa (70; 1:2,000); rabbit anti-RelB (Cell Signaling; 1:100); Rabbit anti-p100/p52 (Cell Signaling; 1:100); and rabbit anti-c-Rel (Santa Cruz Biotechnology; 1:200). Alexa Fluo 488 or 594 conjugated secondary antibodies were then used to detect primary antibodies followed by DAPI counterstaining to reveal the nuclei. RelA-positive umbrella cell nuclei and total umbrella cell nuclei were both recorded and their ratio was presented (number of RelA-positive umbrella cell nuclei in 100 umbrella cell nuclei). Counting of RelA-positive nuclei in experimental and control groups were performed in a blinded fashion. Triple immunofluorescent staining was carried out using goat anti-*E. coli* (Maine Biotechnology Service; 1:300); rabbit anti-RelA (Abcam; 1:2,000) and mouse anti-UPIa (70; 1:300). Corresponding secondary antibodies were those conjugated with Alexa Fluo 488, 594 and 647.

### Whole Mount Bladder Processing, Staining and Confocal Fluorescent Microcopy

Wild-type mouse bladders were transurethrally inoculated with UTI89 (10^8^ CFU) and 3 h after inoculation the bladders were excised, fixed in 4% paraformaldehyde, washed three times in PBS and then permeabilized in 0.5% Triton X-100 for 1 h. After washing in PBS and blocking of non-specific sites in 3% BSA in PBS, the bladders were stained *in situ* with antibodies against *E. coli*, anti-RelA described above and anti-ZO-1 (Invitrogen; 1:1,000) at 4 °C for 24 h, followed by secondary antibodies at 4 °C for 12 h. The whole bladders were mounted on a glass slide and examined with confocal fluorescent microscopy.

### Chemical Inhibition of NF-κB Activity

InSolution™ NF-κB Activation Inhibitor (Millipore), Bay 11-7085 NF-κB inhibitor (Sigma) and InSolution MAPK Inhibitor II (Millipore) were separately diluted in PBS and mixed with UTI89 to a final concentration of 10 μM for each inhibitor. The mixtures were transurethrally inoculated into the mouse bladders (5 mice per inhibitor). The bladders were procured for immunofluorescent staining for RelA 1 h post-inoculation (see above), for detection of intracellular bacterial communities (IBCs) using an anti-E. coli antibody 12 h post-inoculation, and for determination of colony-forming units by bacterial culture 24 h, 3-days and 7-days post-inoculation.

### Statistical Methods

Data were presented as means and standard deviation. The comparison among the experimental groups was performed by one way analysis of variance (ANOVA) with Dunn’s post-test. A p value of less than 0.05 was considered statistically significant.

## Additional Information

**How to cite this article**: Liu, Y. *et al*.Dual ligand/receptor interactions activate urothelial defenses against uropathogenic *E. coli*.*Sci. Rep*.**5**, 16234; doi: 10.1038/srep16234 (2015).

## Supplementary Material

Supplementary Information
