# Supplementary material for: Dual ligand/receptor interactions activate urothelial defenses against uropathogenic E. coli
Source: Sci Rep. 2015 Nov 9;5:16234. doi: 10.1038/srep16234 (PMC4637824; doi:10.1038/srep16234)
Supplement: Supplementary Information [file srep16234-s1.pdf]

Dual ligand/receptor interactions activate urothelial defenses against uropathogenic *E. coli*

Yan Liu, Sylvie Mémet, Ricardo Saban, Xiangpeng Kong,
Pavel Aprikian, Evgeni Sokurenko, Tung-Tien Sun and Xue-Ru Wu

Supplemental Figure 1. Lack of UPEC-mediated activation of non-canonical NF-κB pathway components. A para-aortic lymph node (LN) from a wild-type mouse with skin biting wounds and subsequent systemic bacterial infection and urinary bladders inoculated with UTI89 (1 h and 3 h post-inoculation) were immunohistochemically stained with antibodies against RelB, p100/p52 and c-Rel. Note the nuclear staining (of macrophages) in the LN positive control and the lack of cytoplasmic or nuclear staining of urothelial cells in UPEC-infected bladders. Scar bars: 100 μm (all panels).

Supplemental Figure2. Binding of urothelial nuclear NF-κB on NF-κB responsive elements and urothelial production of pro-inflammatory cytokines. (A) Nuclear protein extracts from PBS- and T1F-UPEC-instilled wild-type mouse urothelial cells were incubated with NF-κB-binding oligonucleotides pre-coated on microtiter wells. The bound NF-κB was detected using direct ELISA with anti-RelA as primary and secondary antibodies followed by colorimetric reading at 450 nm. Note that T1F-UPEC-infected urothelial cells contained much higher levels of NF-κB capable of binding to its consensus binding sequences than those of PBS-instilled bladders (n=5/group; $p<0.001$)). (B) Detection of pro-inflammatory cytokines. Wild-type mice were transurethrally instilled with PBS or with T1F-UPEC strain UTI89 (10^8 cfu) and their urothelia and urine were subject to ELISA detection of TNFα, IL1α, IL1β or IL12. Note the early increase (at 1 h) of TNFα in both urothelia and urine and a relatively later increase (beginning at 6 h) of urothelial and urinary IL1α and IL1β. The further increase of IL1α and the late rise of IL12 at 16 h post-inoculation may reflect the secondary production of the cytokines by inflammatory cells.

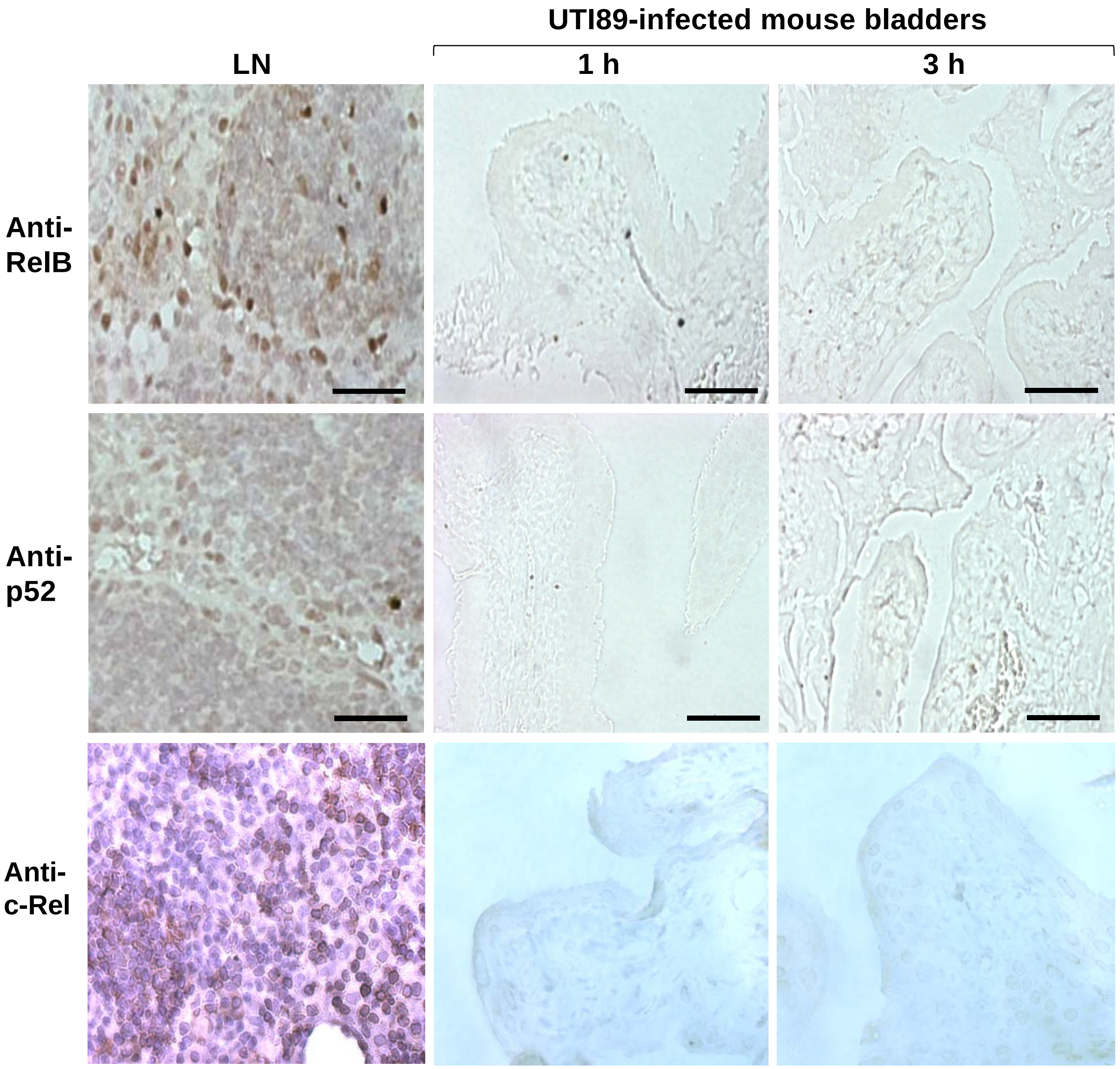
UTI89-infected mouse bladders
LN
1 h
3 h
Anti-
RelB
Anti-
p52
Anti-
c-Rel

Fig. S1

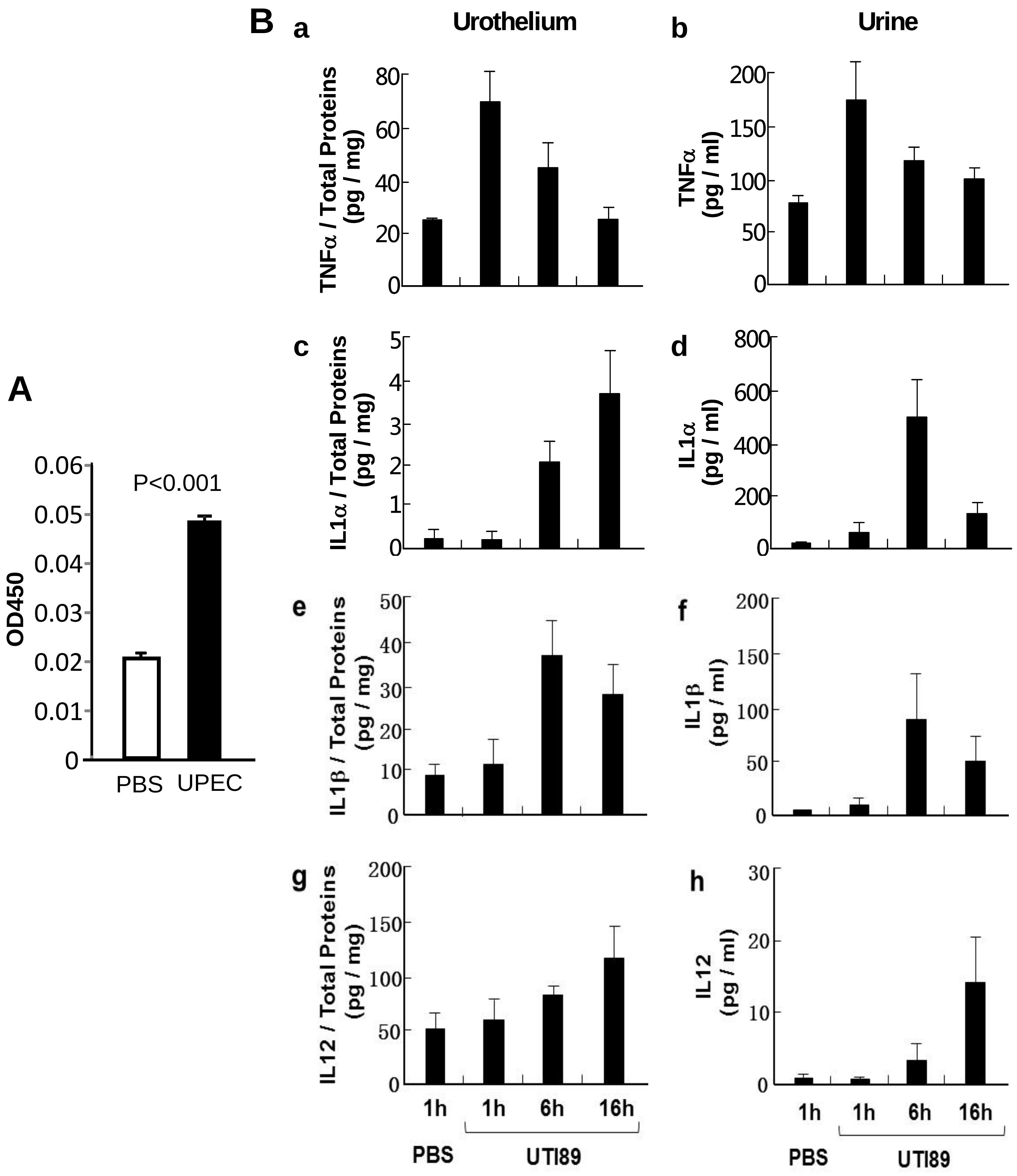
A
OD450
P<0.001
0
0.01
0.02
0.03
0.04
0.05
0.06
PBS
UPEC
B
a
b
Urothelium
Urine
TNFα / Total Proteins (pg / mg)
TNFα (pg / ml)
c
d
IL1α / Total Proteins (pg / mg)
IL1α (pg / ml)
e
f
IL1β / Total Proteins (pg / mg)
IL1β (pg / ml)
g
h
IL12 / Total Proteins (pg / mg)
IL12 (pg / ml)
1h
1h
6h
16h
PBS
UTI89

Fig. S2
